# Model-Based Evaluation of Strategies to Control Brucellosis in China

**DOI:** 10.3390/ijerph14030295

**Published:** 2017-03-12

**Authors:** Ming-Tao Li, Gui-Quan Sun, Wen-Yi Zhang, Zhen Jin

**Affiliations:** 1 Complex Systems Research Center, Shanxi University, Taiyuan 030006, China; limingtao18@126.com (M.-T.L.); sunguiquan@sxu.edu.cn (G.-Q.S.); 2 School of Computer and Information Technology, Shanxi University, Taiyuan 030006, China; 3 Institute of Disease Control and Prevention, Academy of Military Medical Science, Beijing 100071, China; zwy0419@126.com

**Keywords:** brucellosis, dynamic modeling, basic reproduction number, control strategy

## Abstract

Brucellosis, the most common zoonotic disease worldwide, represents a great threat to animal husbandry with the potential to cause enormous economic losses. Brucellosis has become a major public health problem in China, and the number of human brucellosis cases has increased dramatically in recent years. In order to evaluate different intervention strategies to curb brucellosis transmission in China, a novel mathematical model with a general indirect transmission incidence rate was presented. By comparing the results of three models using national human disease data and 11 provinces with high case numbers, the best fitted model with standard incidence was used to investigate the potential for future outbreaks. Estimated basic reproduction numbers were highly heterogeneous, varying widely among provinces. The local basic reproduction numbers of provinces with an obvious increase in incidence were much larger than the average for the country as a whole, suggesting that environment-to-individual transmission was more common than individual-to-individual transmission. We concluded that brucellosis can be controlled through increasing animal vaccination rates, environment disinfection frequency, or elimination rates of infected animals. Our finding suggests that a combination of animal vaccination, environment disinfection, and elimination of infected animals will be necessary to ensure cost-effective control for brucellosis.

## 1. Introduction

Brucellosis, a bacterial disease caused by various *Brucella* species, is one of the most common zoonotic infections globally [1,2,3]. Four *Brucella* species are mainly responsible for the disease: *B. melitensis* in goats and sheep, *B. abortus* typically found in cattle, *B. canis* in dogs, and *B. suis* in swine [4]. Even though these four species of *Brucella* can infect humans, *B. melitensis* remains the major cause of human disease worldwide (and may account for up to 90% of all brucellosis cases [5]). The remaining illnesses are caused by *B. abortus* and *B. suis*, with rare but persisting cases of *B. canis* infections in humans [6]. Although there are a small number of reports of vertical and horizontal transmission between humans [7,8], it is generally acknowledged that human-to-human transmission of the infection is a very rare event [9].

Humans become infected typically through consumption of the unpasteurized products contaminated by the bacterial agent, and to a lesser extent, contact with infected animals. Consequently, brucellosis in humans is strongly linked to the management of infected animals and ingestion of unpasteurized dairy products [10]. According to the length and severity of symptoms, the disease in humans is arbitrarily classified as acute (less than 8 weeks), subacute (from 8 to 52 weeks), or chronic (more than 1 year) [9]. The disease is commonly underreported, misdiagnosed and once chronic disease develops, it is resistant to treatment, which consists of antibiotics for long periods [11,12,13]. Mortality is reported to be negligible, but the illness can persist for several years. Though less severe in animals, brucellosis can cause economic losses by adversely affecting reproduction and fertility, survival of newborns, and milk yields [14,15].

Human brucellosis matches the regions of the world with high levels of animal infection endemicity: the Mediterranean basin, Middle East, Western Asia, Africa, and South America [3] where hundreds of thousands of new cases are reported annually [9,16]. In developed countries, control of animal brucellosis has been successfully achieved. However, many of these control options are less achievable in developing countries [17]. In China, human brucellosis is a class B notifiable infectious disease, and information regarding each confirmed case must be reported to the Chinese Center for Disease Control and Prevention (CCDC) through the National Notifiable Disease Surveillance System (NNDSS) since 2004 [18]. Although many measures based on the control programs for brucellosis have been set up, the brucellosis-positive rate in humans has increased significantly in recent years [19]. It is believed that reduced awareness of the disease and concordant reductions in surveillance and vaccination rates has led to an overall rise in human cases [20]. Hence, reconsidering the use of animals vaccination to reduce susceptibility to brucellosis infection has become urgent.

Mathematical modeling has the potential to analyze the mechanisms of transmission and the complexity of epidemiological characteristics of infectious diseases, and can indicate new approaches to prevent and control future epidemics [21,22]. In recent years, several mathematical modeling studies have reported on the transmission of brucellosis [23,24,25,26,27,28,29,30]. However, these earlier models have mainly focused on the spread of brucellosis through using statistical methods or the theoretical brucellosis dynamic model. Only Hou et al. [25] and Li et al. [26] studied the underlying dynamics of brucellosis transmission between sheep and human in Inner Mongolia, China, and explicitly quantified levels of transmission between *Brucella* of environment and individuals. But model-based evaluations do not yet exist for strategising the control of brucellosis in mainland China and other provinces. In this paper, we explored the utility of three different dynamic mathematical models to explain the incidence of brucellosis from 2004 to 2014, and the best-fit model was selected by using Akaike information criterion. The best-fit model is then used to investigate the potential for future outbreaks, and to estimate the national-level basic reproduction number as well as the more local-level outbreak thresholds for the eleven provinces with high case numbers (including Inner Mongolia, Shanxi, Heilongjiang, Hebei, Xinjiang, Jilin, Henan, Liaoning, Shaanxi, Shandong and Ningxia), and to provide new insights into the epidemiology of brucellosis, including province-level control targets required to achieve elimination.

## 2. Materials and Methods

### 2.1. Data Collection

**Figure 1:**
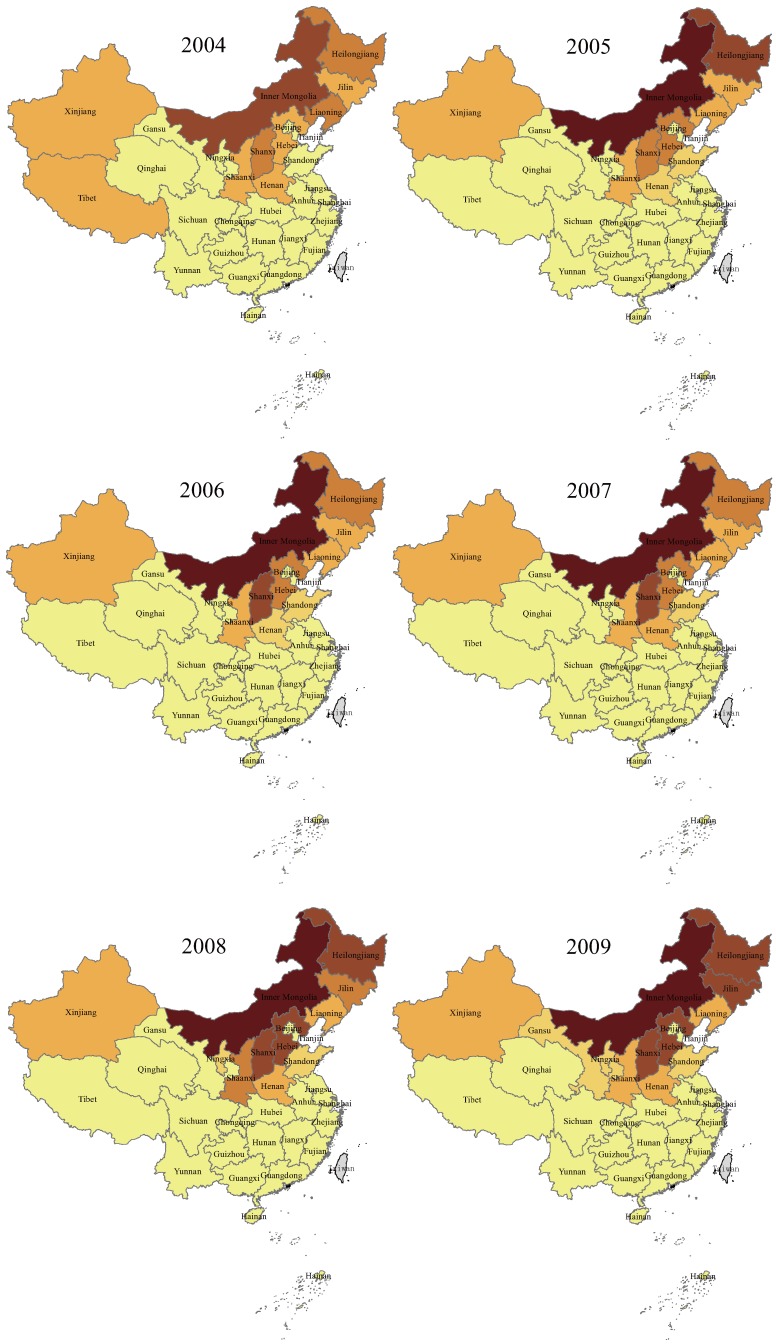

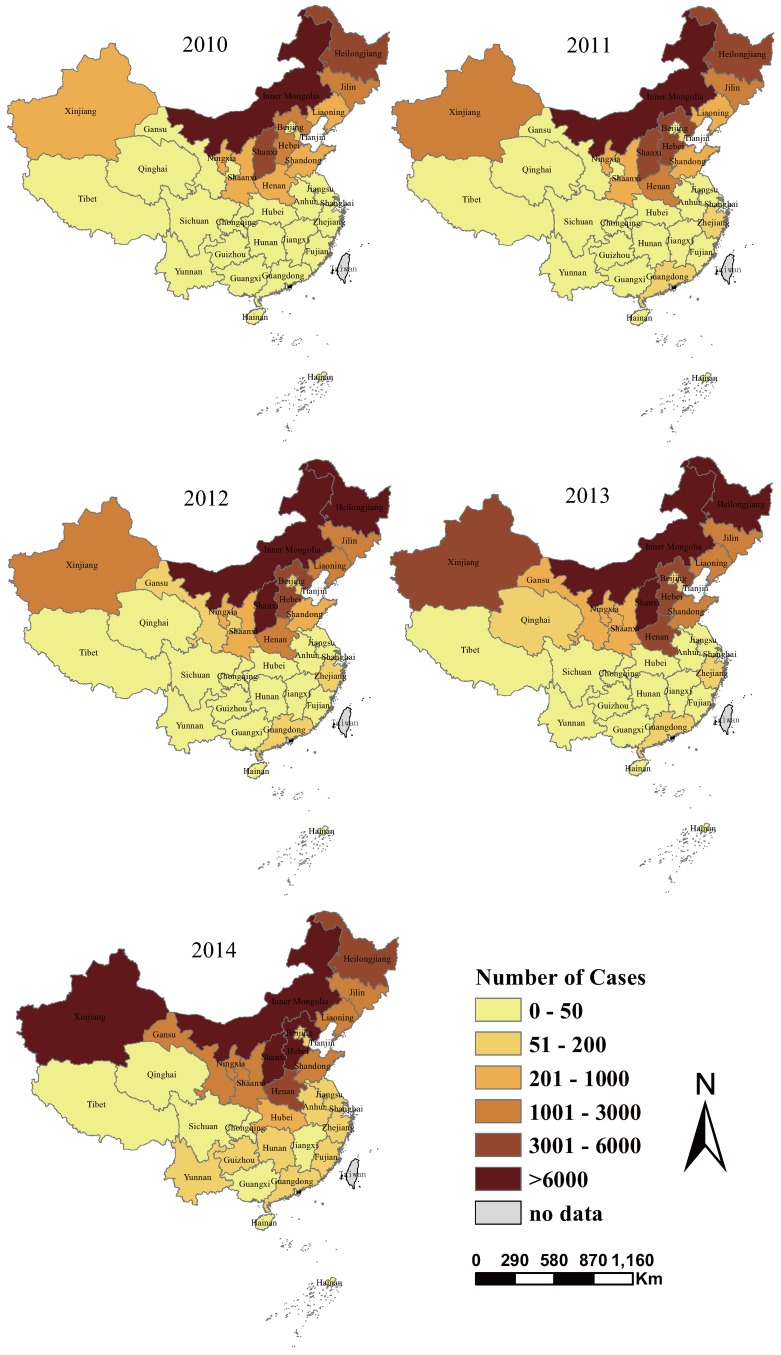
Spatiotemporal distribution of annual human brucellosis cases, by province, in China, 2004–2014.

The annual and cumulative reported human brucellosis cases of mainland China and the selected 11 provinces with high case numbers from 2004 to 2014 were obtained from the National Notifiable Disease Surveillance System (NNDSS). The spatiotemporal distribution map presented in Figure 1 showed that human brucellosis was widely distributed in northern, northeastern, and western China. Elsewhere, cases are more sporadic. The 11 provinces with high case numbers include Inner Mongolia, Shanxi, Heilongjiang, Hebei, Xinjiang, Jilin, Henan, Liaoning, Shaanxi, Shandong and Ningxia; these provinces accounted for > 98.5% of the national reported cases. During 2004–2014, a total of 344,351 new cases were reported in mainland China, with the annual incidence of human brucellosis increasing. In 2004, only 21 provinces reported 11,472 new cases of human brucellosis, but the epidemic regions expanded to 30 provinces of China (except for Hong Kong, Macao, Taiwan, and Tibet) with 57,222 cases reported in 2014.

### 2.2. Mathematical Model

**Figure 2:**
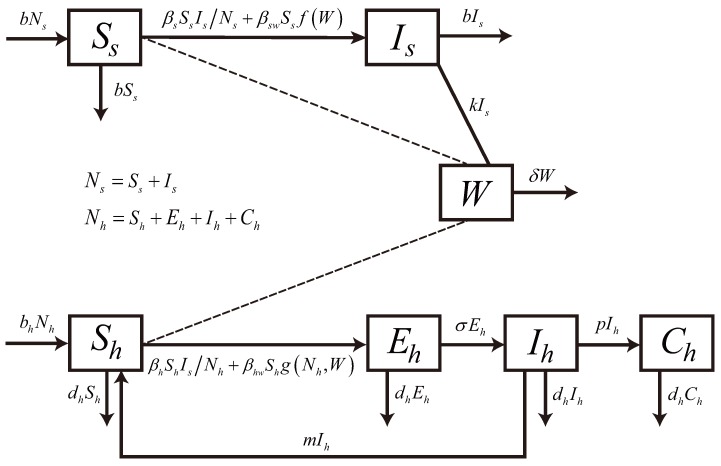
Transmission diagram on the dynamical transmission of brucellosis.

Brucellosis can be transmitted to individuals through direct contact with infected animals or indirect transmission by *Brucella* in the environment, which contains contaminated forage, water, grass, liquids, products and the uterine fluids from infected animals [26]. Environment-to-individual transmission is defined as a indirect transmission process with a high infectious dose, resulting from contact with *Brucella* in the environment. In contrast, individual-to-individual transmission is assumed to be a direct transmission process, with a lower infectious dose. In mainland China, *B. melitensis* (sheep-type *Brucella*) is the predominant pathogen associated with large outbreaks [31]. The result of Li et al. [20] also indicated that sheep and goats probably were the main animal hosts transmitting the diseases to humans in northern, northeastern and western China. Hence, sheep and goats were considered and called sheep population in the model. There are some assumptions for our model: (1) Brucellosis in the exposure period is hardly detected, and animals in this period can also infect susceptible sheep and humans. Hence, we ignored the exposed period in the sheep population; (2) In China in the 20th century, the control measure for brucellosis was mainly vaccinating livestock [31], but there are few reports on animal vaccination in the first fifteen years of 21st century. Therefore, animal vaccination is also ignored; (3) There is no data reporting human-to-human transmission of brucellosis, so the rate of human-to-human and human-to-animal transmission was ignored. There are also some other assumptions on our model, which are demonstrated in the flowchart (see Figure 2).

In this study, the brucellosis model classified the human population (denoted by $N_{h}$) into susceptible $S_{h}$, exposed $E_{h}$, acute infectious $I_{h}$, and chronic infectious $C_{h}$ compartments. Human population birth and death rates were included as $b_{h}$ and $d_{h}$, respectively. In China, the human birth rate has been greater than the death rate in recent years. The human host incubation period is 1/σ years, and the incubation period of human brucellosis is about two weeks [32], so the clinical outcome rate of exposed people is σ=26 per year. Acute infections progress at the rate *p* into chronic infections and revert to susceptible at rate *m*. The population of sheep, denoted $N_{s}$, were separated into either susceptible $S_{s}$ and infectious $I_{s}$ compartments. From the China Animal Husbandry Statistical Yearbook [33], one could calculate that the yearly breeding stock deviation of the sheep population in mainland China was less 5 percent, so sheep recruitment and slaughter rates, listed as *b*, were set to balance each other. Let *W* denote the density of *Brucella* in the environment. Animals with brucellosis shed *Brucella* in the environment at rate *k* and environmental *Brucella* had a mortality rate of *δ*, and environmental shedding mainly includes the contaminated products by infected animals. Susceptible animals acquire brucellosis either through direct transmission at rate $\beta_{s}S_{s}\frac{I_{s}}{N_{s}}$ or by ingesting environmental *Brucella* at transmission rate $\beta_{sw}S_{s}f\left(W\right)$. Susceptible humans acquire brucellosis through direct contact with infected animals or indirect transmission by environmental *Brucella* at rates $\beta_{h}S_{h}\frac{I_{s}}{N_{h}}$ and $\beta_{hw}S_{h}g\left(N_{h},W\right)$, respectively. In this study, the standard incidence and general incidence rates were used for direct contact transmission and indirect transmission, respectively. Hence, the brucellosis model is described by the following ordinary differential equations:

(1) $$\left\{\begin{array}{c} \frac{dS_{s}}{dt}=bN_{s}-\beta_{s}S_{s}\frac{I_{s}}{N_{s}}-\beta_{sw}S_{s}f\left(W\right)-bS_{s},\\ \frac{dI_{s}}{dt}=\beta_{s}S_{s}\frac{I_{s}}{N_{s}}+\beta_{sw}S_{s}f\left(W\right)-bI_{s},\\ \frac{dW}{dt}=kI_{s}-\delta W,\\ \frac{dS_{h}}{dt}=b_{h}N_{h}+mI_{h}-\beta_{h}S_{h}\frac{I_{s}}{N_{h}}-\beta_{hw}S_{h}g\left(N_{h},W\right)-d_{h}S_{h},\\ \frac{dE_{h}}{dt}=\beta_{h}S_{h}\frac{I_{s}}{N_{h}}+\beta_{hw}S_{h}g\left(N_{h},W\right)-\sigma E_{h}-d_{h}E_{h},\\ \frac{dI_{h}}{dt}=\sigma E_{h}-pI_{h}-mI_{h}-d_{h}I_{h},\\ \frac{dC_{h}}{dt}=pI_{h}-d_{h}C_{h},\\ N_{s}=S_{s}+I_{s},N_{h}=S_{h}+E_{h}+I_{h}+C_{h}. \end{array}\right.$$

All parameters of Equation (1) are assumed to be nonnegative. The mathematical properties of the brucellosis model including calculations for the basic reproduction number and global stability of the equilibrium are given in **Section 2** Dynamical behavior (Supplementary Material).

## 3. Numerical Results

Due to the general incidence rates of indirect transmission in Equation (1), multiple models can be created. To evaluate the situation of brucellosis in some provinces and the whole country (mainland China), we need to choose the most appropriate functional form for indirect brucellosis transmission i.e., a comparison could be made between transmission determined by a mass action incidence rate, saturating incidence rate and standard incidence rate. Case 1. Standard incidence rate: $\beta_{sw}S_{s}f\left(W\right)=\beta_{sw}S_{s}\frac{W}{N_{s}},\beta_{hw}S_{h}g\left(N_{h},W\right)=\beta_{hw}S_{h}\frac{W}{N_{h}}$. Case 2. Saturating incidence rate: $\beta_{sw}S_{s}f\left(W\right)=\beta_{sw}S_{s}\frac{W}{\varepsilon +W},\beta_{hw}S_{h}g\left(N_{h},W\right)=\beta_{hw}S_{h}\frac{W}{\varepsilon +W}$. Case 3. Mass action incidence rate: $\beta_{sw}S_{s}f\left(W\right)=\beta_{sw}S_{s}\frac{W}{M},\beta_{hw}S_{h}g\left(N_{h},W\right)=\beta_{hw}S_{h}\frac{W}{M}$. Here, *ε* and *M* are scaling factors for the *brucella* concentration in the environment.

To determine the most appropriate functional form, we firstly fitted each Case of model to the annual infection data on numbers of national human brucellosis cases from 2004 to 2014. Literature reviews facilitated epidemiological parameter values for brucellosis (including extrinsic incubation period of human brucellosis, transfer rate from acute infections to chronic infections and susceptible populations, *Brucella* shedding rate by infected animals and the decaying rate of *Brucella* in the environment) are given in Table S1 (Supplementary Material). From the China Animal Husbandry Statistical Yearbook [33], we can find that the value of sheep recruitment and slaughtering rate is b=0.9026, (0.8531–0.9521) for mainland China. From the China Statistical Yearbook [34], one can obtain the average values and corresponding 95% confidence intervals of the human birth rate $b_{h}$ and death rate $d_{h}$ from 2004 to 2014 for mainland China, which are $b_{h}=0.012098$, (0.0119863–0.0122097) and $d_{h}=0.006937$, (0.0067422–0.0071318), respectively.

### 3.1. Parameter Estimation

For the initial values of the model in mainland China, the susceptible sheep and human population, the number of cases of human brucellosis infection can be directly obtained from [33,34], which are $S_{s}\left(0\right)=2.85\times 10^{8}$, $S_{h}\left(0\right)=1.29998\times 10^{9}$, and $I_{h}\left(0\right)=11472$, respectively. However, the number of infected animals, the density of *Brucella* in the environment, and the number of cases of exposure need estimates. The number of annual cases of human brucellosis infection in mainland China were used to estimate the transmission rate of $\beta_{s}$, $\beta_{sw}$, $\beta_{h}$ and $\beta_{hw}$. We firstly fixed the human indirect transmission rate and assumed that $\beta_{hw}=0.5$, because there might be high correlations between $\beta_{s}$, $\beta_{sw}$, $\beta_{h}$ and $\beta_{hw}$. Then, we estimated the initial values of model and transmission rate of $\beta_{s}$, $\beta_{sw}$, $\beta_{h}$ by using the least-squares fitting routine fminsearch in MATLAB. The least-square estimation is adopted here to find the parameter values to minimize the objective function:

$$L=\frac{1}{n}\sum_{t=1}^{n}{\left(Y\left(t\right)-y\left(t\right)\right)}^{2},$$

where Y(t) is the theoretical number of human infection cases, y(t) is the actual reported human cases at year *t*, and *n* is the number of reported data. Finally, using the obtained values from MATLAB, the resulting estimates for parameters $\beta_{s}$, $\beta_{sw}$, $\beta_{h}$ of mainland China could be generated (independently for all three incidence functions) through DEDiscover software (DEDiscover is a general-purpose tool to perform simulation, parameter estimation and statistical analysis for any problem that can be expressed as a set of differential Equations [35]), and listed in Table 1.

### 3.2. Model Selection

**Figure 3:**
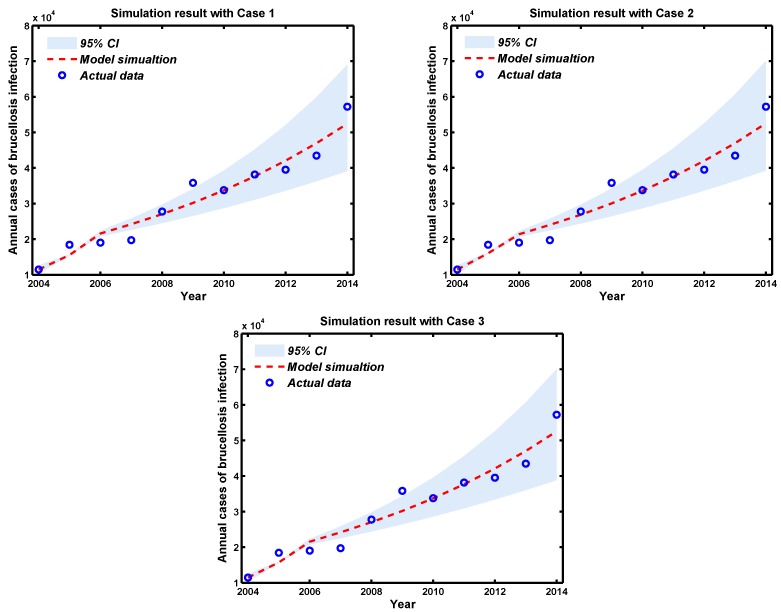
Brucellosis model fitting for the annual cases of human brucellosis infection with different Cases. The light grey shaded area shows the 95% confident interval (CI) for all 1000 simulations, and the blue circles mark the reported data for human brucellosis cases. Let x(t) represent annual cases of brucellosis infection, and x(t)=X(t)−X(t−1), where $\frac{dX(t)}{dt}=\sigma E_{h}\left(t\right)$.

Figure 3 shows the comparison between theoretical and annual cases of human brucellosis infections in mainland China are plotted along with the 95% percent interval for 1000 simulation outputs from the alternative models Cases 1–3. From Figure 3, we can conclude that in three cases, there is no obvious difference in the simulation results. In order to check the adequacy of the model, we use the method of Akaike information criterion (AIC) [36,37,38], to compare the validity of the three results and choose the best model from multiple competing models. Since the ratio of the number of data points to the number of parameters fitted is less than 40, we need to compute the $AIC_{c}$ for each model. $AIC_{c}$ values corresponding to three Cases are $AIC_{c1}=181.4021$, $AIC_{c2}=186.8334$, $AIC_{c3}=186.6243$, and the model with the lowest $AIC_{c}$ is Case 1. Moreover, Δ $AIC_{c2}=AIC_{c2}-AIC_{c1}=5.3004>4$ and Δ $AIC_{c3}=AIC_{c3}-AIC_{c1}=5.2222>4$, which imply that assumptions Case 2 and Case 3 are considered as the model with less support. In a word, the model selection results show that Case 1, the standard incidence function, is consistently the best model to be used for inference.

For 11 selected provinces with high case numbers, we also fitted each model for the yearly data to determine the most appropriate functional form. For Inner Mongolia and Jilin, we noted significant reductions in case numbers following 2011 and 2010, respectively. The causes for the decrease in these two provinces are prevention and control strategies including animal vaccination and elimination of the infected animals. For the Heilongjiang and Shannxi provinces, the cumulative number of brucellosis cases was used to give the estimation. Also from the China Statistical Yearbook [34] and China Animal Husbandry Statistical Yearbook [33], one can obtain the demographic parameter values (including human population birth rate $b_{h}$ and death rates $d_{h}$, sheep recruitment and slaughtering rate *b*) and their corresponding 95% confidence intervals for these 11 provinces, listed in Table S2 (Supplementary Material). Fixing the human indirect transmission rate as $\beta_{hw}=0.5$, the estimates of $\beta_{s}$, $\beta_{sw}$, $\beta_{h}$ for these 11 provinces are also obtained by using DEDiscover software, and shown in Table S3 (Supplementary Material). The alternative model structures were further compared through assessing their fit to the eleven provinces with high case numbers, which also show that the standard incidence function was the best model to be used. Hence, the following model will be used for further simulation:

(2) $$\begin{array}{c} \left\{\begin{array}{c} \frac{dS_{s}}{dt}=bN_{s}-\beta_{s}S_{s}\frac{I_{s}}{N_{s}}-\beta_{sw}S_{s}\frac{W}{N_{s}}-bS_{s},\\ \frac{dI_{s}}{dt}=\beta_{s}S_{s}\frac{I_{s}}{N_{s}}+\beta_{sw}S_{s}\frac{W}{N_{s}}-bI_{s},\\ \frac{dW}{dt}=kI_{s}-\delta W,\\ \frac{dS_{h}}{dt}=b_{h}N_{h}+mI_{h}-\beta_{h}S_{h}\frac{I_{s}}{N_{h}}-\beta_{hw}S_{h}\frac{W}{N_{h}}-d_{h}S_{h},\\ \frac{dE_{h}}{dt}=\beta_{h}S_{h}\frac{I_{s}}{N_{h}}+\beta_{hw}S_{h}\frac{W}{N_{h}}-\sigma E_{h}-d_{h}E_{h},\\ \frac{dI_{h}}{dt}=\sigma E_{h}-pI_{h}-mI_{h}-d_{h}I_{h},\\ \frac{dC_{h}}{dt}=pI_{h}-d_{h}C_{h},\\ N_{s}=S_{s}+I_{s},N_{h}=S_{h}+E_{h}+I_{h}+C_{h} \end{array}\right. \end{array}$$

### 3.3. Fitting Results

**Figure 4:**
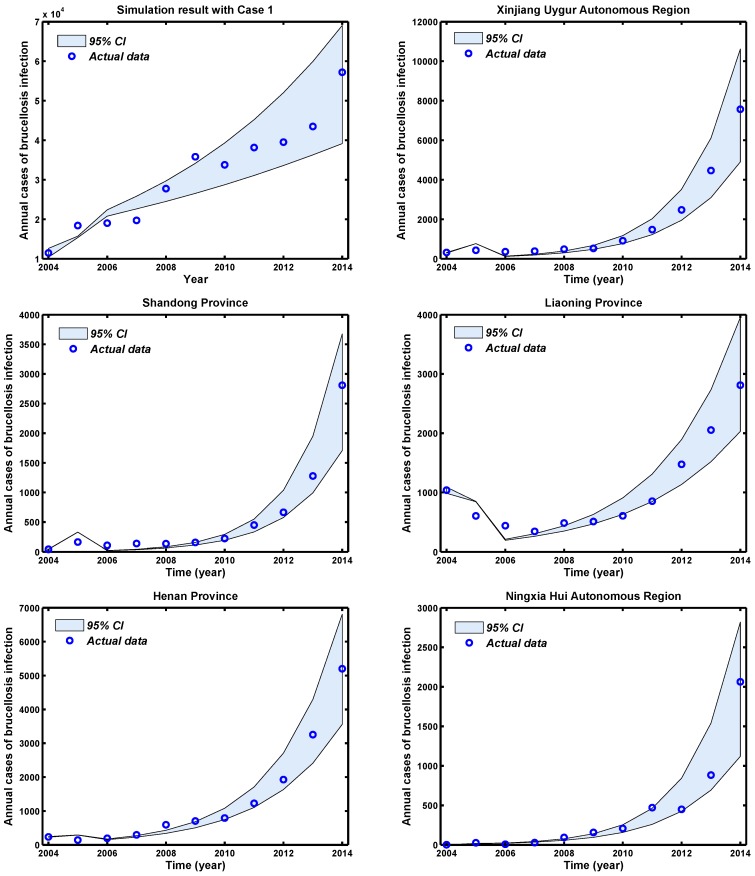

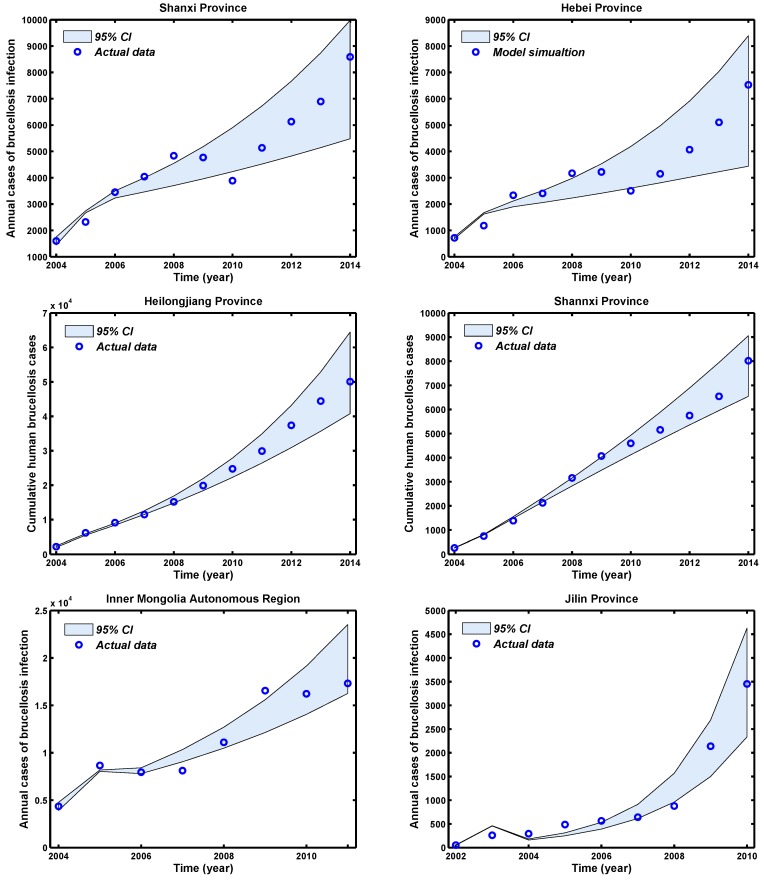
Brucellosis model fitting for cases of human brucellosis infection in mainland China and 11 selected provinces. The light grey shaded area shows the 95% CI for all 1000 simulations, and the blue circles mark the reported data for human brucellosis cases.

The provinces with the highest risk of brucellosis incidence in the country are very distinct in many ways, for example with respect to animal habitat and animal and human population densities. The process was initially conducted for mainland China (total) and then repeated for each of the 11 provinces with high case numbers. Through using the available parameters in Tables S1 and S2 (Supplementary Materials), and the estimated parameters in Table S3 (Supplementary Materials), Monte Carlo simulation runs were then conducted to assess the data fitting of the cases of brucellosis infection with mainland China and 11 provinces with high case numbers, and the plots are shown in Figure 4. The 95% percent interval for all 1000 passing simulation trajectories show that there is a good match between the reported data and the theoretical prediction of Equation (2).

### 3.4. Estimation of Basic Reproduction Numbers

The basic reproduction number provides useful guidelines for the prevention and control strategies for epidemics. For Equation (2), the basic reproduction number is:

(3) $$\begin{array}{c} R_{0}=\frac{\beta_{s}}{b}+\frac{k\beta_{sw}}{b\delta }=R_{0}^{i}+R_{0}^{e} \end{array}$$

where $R_{0}^{e}=\frac{k\beta_{sw}}{b\delta }$ and $R_{0}^{i}=\frac{\beta_{s}}{b}$ are partial reproduction numbers due to environment-to-individual transmission and individual-to-individual transmission, respectively. Using k=15,δ=3.6, the average value of *b* and relevant parameter values in Table S3 (Supplementary Materials), and the corresponding values of $R_{0}^{i}$, $R_{0}^{e}$ and $R_{0}$ for the 11 selected provinces and mainland China are given in Table 2. These quantities of $R_{0}>1$ obtained for the 11 selected provinces and mainland China imply that future epidemics are highly likely, unless effective control measures are put in place. The local basic reproduction numbers of the provinces with an obvious increase in incidence (including Xinjiang, Henan, Liaoning, Shandong, Jilin and Ningxia) are much larger than average for the whole country. Also in these six provinces, the partial reproduction numbers for environment-to-individual transmission are much larger than for individual-to-individual transmission.

### 3.5. Modeling Interventions

In recent years, control of animal brucellosis has been successfully achieved in the developed world through the combination of vaccination and test-and-slaughter programs, coupled with effective disease surveillance and animal movement control [3]. In developing countries, however, control by test-and-slaughter and animal vaccination is hardly achievable because of limited resources to indemnify farmers [16]. However, in China, the vaccination against brucellosis of domestic animals has resulted in a rapid decline in the incidence of brucellosis in animals and humans in the 1980s and 1990s [31]. Hence, we believe that brucellosis can be controlled in China if the coverage of vaccination is broad enough. The same conclusion was made in [14], and also claimed that if vaccination rate is too high it may be impossible to eradicate brucellosis as it has been the case in low-income and middle-income countries like India, Mongolia, Mexico and Russia et al. We will study the impact of animal vaccination, elimination of infected animals, and environment disinfection for brucellosis control in China.

Animal vaccination can cause a rapid decline in the incidence of brucellosis in animals and humans [31]. For the control measure of animal vaccination, the assumption is that *s* is the vaccine efficacy level, and *v* is the coverage of the vaccination programme (i.e., the proportion of the population vaccinated). Then, the vaccination programme reduces the reproduction number to the value $R_{0}\left(1-v\cdot s\right)$. From this it follows that the minimum coverage of animals vaccination rate for eradication brucellosis is given by:

(4) $$\begin{array}{c} v\geq s^{-1}\left(1-R_{0}^{-1}\right). \end{array}$$

A recent study [25] conducted in Inner Mongolia demonstrated that the vaccine effectively protects approximately 82% of sheep i.e., we assume s=0.82.

The control measure of quarantine, separation and elimination of the infected animals for brucellosis can also be incorporated depending on the animals and regions. The assumption is that *α* is the infected-animal removal rate, and the control reproduction number is $R_{0}\frac{b}{b+\alpha }$. Hence, the brucellosis epidemic can be controlled only with this measure with a minimum coverage of:

(5) $$\begin{array}{c} \alpha \geq b(R_{0}-1). \end{array}$$

In our model, the density of *brucella* in environment is denoted by *W*. Hence, the disinfection of environment can be considered another measures for brucellosis. The assumption is that *l* is the disinfection frequency and the unit is time, *m* is the effective disinfection rate each time, and the control reproduction number of disinfection is $R_{0}^{i}+R_{0}^{e}\frac{\delta }{\delta +lm}$. Hence, the minimum disinfection frequency for controlling brucellosis is described as:

(6) $$\begin{array}{c} l\geq \frac{\delta }{m}\frac{R_{0}^{i}+R_{0}^{e}-1}{1-R_{0}^{i}}. \end{array}$$

There is no study on the effective disinfection rate each time. In the following calculations we assume that m=0.5.

The minimum vaccination coverage rate, removal rate, and disinfection frequency required to control brucellosis epidemics for the 11 selected provinces and mainland China are presented in Table 3. The outbreak of brucellosis in mainland China could be controlled by animal vaccination, or elimination of the infected animals, or environment disinfection, as long as the measure was strict: the minimum vaccination coverage rate was 0.1478, the minimum removal rate of infected animals was 0.1245, or the minimum disinfection frequency was three times per year. However, this was highly heterogeneous among the 11 provinces with high case numbers: the vaccination coverage necessary for control ranged from 0.0271 to 0.5549 and the removal rate ranged from 0.0154 to 0.7108, while the disinfection frequency ranged from 1 to 19 times per year. Furthermore, we conclude that brucellosis may be easily controlled by a combination of animal vaccination, environment disinfection, and elimination of infected animals.

## 4. Discussion

The re-emergence of brucellosis in recent years represents one of the major public health threats in mainland China. Since the beginning of the 21st century, the number of human brucellosis cases has dramatically increased throughout China and the number of human cases has reached a historic high with 57,222 cases reported in 2014. In the period 2004–2014, the human brucellosis epidemic was largely confined to the northern provinces (mainly including Inner Mongolia, Shanxi, Heilongjiang, Hebei, Xinjiang, Jilin, Henan, Liaoning, Shaanxi, Shandong and Ningxia; see Figure 1) where animal husbandry is commonly practiced, and thus the risk of infection is higher. Epidemiological reports on human brucellosis have previously been associated with animal habitat, occupation, host density, socioeconomic status, travel and immigration [39,40,41,42,43]. Although many measures based on the control programs for brucellosis have been set up, the brucellosis-positive rate in animals has increased significantly, along with an increase in human brucellosis cases in recent years. Increase in animal feeding, lack of immunization and animal quarantine, and frequent trading have been implicated as the key risk factors for the dramatic increase in brucellosis incidence in the past decade [19].

To investigate the underlying dynamics of brucellosis transmission in mainland China including its provinces with high prevalence, a mathematical model was constructed and fit to human incidence data. Under the general biological assumptions, we have given the formula of the basic reproduction number. We have also proven the global stability of the disease-free equilibrium when $R_{0}<1$, and the global stability of the endemic equilibrium when $R_{0}>1$. The model was then used to explore the magnitude of control and prevention measures necessary to block brucellosis transmission. By comparing the results of three models using national human disease data and 11 provinces with high case numbers, the best-fitted model with standard incidence was used to investigate the potential for future outbreaks. The estimation of the reproductive numbers (Table 2) shows that future epidemics in 11 selected provinces and mainland China are highly likely, unless effective control measures are put in place. The local basic reproduction number of the provinces with an obvious increase in incidence (including Xinjiang, Henan, Liaoning, Shandong, Jilin and Ningxia) are much larger than average for the whole country. Our model suggests that brucellosis can be controlled through animal vaccination, environmental disinfection, or elimination of infected animals. Hence, much remains need to be done for the local and provincial CDCs aiming to reach the goal of controlling human and animal brucellosis in China.

The current study suffers from several limitations. One limitation of the current model is that the mainland China and 11 provinces with high case numbers were treated as homogenous and well-mixed whereas in reality, this is likely not the case. To try to control for this heterogeneity, we did separately model the 11 provinces with the highest incidence, but even within those provinces there will be areas with higher local transmission than others. There also exists a strong age structure, sex structure and occupational association with human brucellosis [20], and therefore a spatially explicit age-structured model would be studied in the future. Finally, the economic costs of different control measures also needed to be considered for future intervention targeting with different regions.

## 5. Conclusions

Nevertheless, the brucellosis dynamic model developed in this study could increase the understanding of the spread and control of the disease, identify the mechanisms influencing transmission dynamics, and reflect the current trend in the incidence of human brucellosis in mainland China. Our estimates of the basic reproduction numbers for selected 11 provinces (in addition to the country on a whole) can quantify the magnitude of human brucellosis in mainland China, with these initial estimates conveying important information about the prospects for effective control measures. Our investigation may be also potentially helpful to strategise control for the prevention and surveillance of brucellosis beyond China, in other endemic countries.

## Figures and Tables

**Table 1:** Model selection table for mainland China.

	Case 1	Case 2	Case 3
$\beta_{s}$	0.5583 (0.5522–0.5643)	0.7401 (0.7328–0.7475)	0.7580 (0.7545–0.7615)
$\beta_{sw}$	0.1125 (0.1086–0.1165)	0.2761 (0.2713–0.2809)	0.2798 (0.2788–0.2808)
$\beta_{h}$	0.0676 (0.0662–0.0691)	0.0942 (0.0924–0.0960)	0.0752 (0.0727–0.0777)
*ε* ($10^{9}$)	-	1.1846 (1.1844–1.1848)	-
*M* ($10^{9}$)	-	-	1.2911 (1.2910–1.2912)
AIC	177.9735	180.0358	179.9576
$AIC_{c}$	181.4021	186.7025	186.6243
Δ $AIC_{c}$	0	5.3004	5.2222

**Table 2:** Estimated values of $R_{0}^{i}$, $R_{0}^{e}$, $R_{0}$ and their 95% confidence intervals.

	$R_{0}^{i}$	95% CI	$R_{0}^{e}$	95% CI	$R_{0}$	95% CI
Mainland China	0.6185	(0.6118-0.6252)	0.5193	(0.5013–0.5475)	1.1379	(1.1131–1.1727)
Xinjiang	0.2864	(0.1916–0.3812)	1.5131	(1.4110–1.6157)	1.7995	(1.6025–1.9970)
Shandong	0.4347	(0.2987–0.5706)	1.1812	(1.0474–1.3179)	1.6159	(1.3461–1.8885)
Liaoning	0.1886	( 0.1148–0.2625)	1.2482	(1.1835–1.3125)	1.4369	(1.2983–1.5750)
Henan	0.2213	(0.1694–0.2733)	1.3132	(1.2024–1.2789)	1.5346	(1.3719–1.6973)
Ningxia	0.4417	(0.2469–0.6365)	1.2931	(1.2013–1.8051)	1.8348	(1.4482–2.4417)
Shanxi	0.7623	(0.7120–0.8127)	0.4456	(0.3579–0.5324)	1.2079	(1.0699–1.3451)
Hebei	0.5012	(0.4341–0.5683)	0.6208	(0.5210–0.7203)	1.1220	(0.9550–1.2886)
Heilongjiang	0.4808	(0.4039–0.5577)	0.6946	(0.6183–0.7714)	1.1754	(1.0222–1.3292)
Shaanxi	0.6708	(0.5869–0.7546)	0.3519	(0.2595–0.4443)	1.0227	(0.8464-1.1990)
Inner Mongolia	0.6135	(0.5896–0.6375)	0.5854	(0.5475–0.6233 )	1.1989	(1.1371–1.2608)
Jilin	0.6974	(0.4775–0.9174)	1.0782	(0.9005–1.2559 )	1.7756	(1.3780–2.1734)

**Table 3:** Estimates of minimum vaccination coverage rate *v*, removal rate *α*, and disinfection frequency *l*.

	Vaccination Rate	Removal Rate	Disinfection Frequency (${\mathrm{Time}}^{-1}$)
Mainland China	0.1478	0.1245	3
Xinjiang	0.5418	0.6686	9
Shandong	0.4648	0.8132	8
Liaoning	0.3708	0.4331	4
Henan	0.4248	0.5770	5
Ningxia	0.5549	0.7108	11
Shanxi	0.2099	0.1058	7
Hebei	0.1326	0.1512	2
Heilongjiang	0.1820	0.1379	3
Shaanxi	0.0271	0.0154	1
Inner Mongolia	0.2023	0.2034	4
Jilin	0.5327	0.5847	19

## Supplementary Materials

The following are available online at www.mdpi.com/1660-4601/14/3/295/s1, Table S1: Constant parameters description and values ($year^{-1}$), Table S2: Values of *b*, $b_{h}$ and $d_{h}$ and 95% confidence intervals ($year^{-1}$), Table S3: Estimated values of $\beta_{s}$, $\beta_{sw}$ and $\beta_{h}$ with their 95% confidence intervals ($year^{-1}$). Dynamical behavior.
