# Supplementary material for: Model-Based Evaluation of Strategies to Control Brucellosis in China
Source: Int J Environ Res Public Health. 2017 Mar 12;14(3):295. doi: 10.3390/ijerph14030295 (PMC5369131; doi:10.3390/ijerph14030295)
Supplement: Supplementary file 1 [file ijerph-14-00295-s001.pdf]

**Supplementary Material: Model-Based Evaluation of Strategies to Control Brucellosis in China**

Ming-Tao Li, Gui-Quan Sun, Wen-Yi Zhang, Zhen Jin

# 1 Model parameters and values

**Constant parameters.** Table S1 shows the epidemiological parameter values for brucellosis in literature. The unit of the model parameters is one year. The incubation period of human brucellosis is about two weeks [1], so the clinical outcome rate for exposed people is $\sigma = 26$ per year. From the China Statistical Yearbook [2] and the China Animal Husbandry Statistical Yearbook [3], one can obtain the demographic parameter values (including human populations birth rate $b_h$ and death rates $d_h$, sheep recruitment and slaughter rate $b$) for mainland China and the 11 provinces with the highest incidence for human brucellosis listed in Table S2.

Table S1: Constant parameters description and values ($year^{-1}$).

| Description | Notation | Value | Reference |
|---|---|---|---|
| Extrinsic incubation period of human brucellosis | $\frac{1}{\sigma}$ | $\frac{1}{26}$ | [1] |
| Transfer rate from acute infections to chronic infections | $p$ | 0.6 | [4] |
| Transfer rate from acute infections to susceptible populations | $m$ | 0.4 | [4] |
| *Brucella* shedding rate by infected animals | $k$ | 15 | [4] |
| The decaying rate of *Brucella* in the environment | $\delta$ | 3.6 | [4] |

Table S2: Values of $b$, $b_h$ and $d_h$ and 95% confidence intervals ($year^{-1}$).

| | $b$ 95% CI | $b_h$ 95% CI ($10^{-3}$) | $d_h$ 95% CI ($10^{-3}$) |
|---|---|---|---|
| Mainland China | 0.9026 (0.8531-0.9521) | 12.098 (11.9863-12.2097) | 6.937 (6.7422-7.1318) |
| Xingjiang | 0.8363 (0.7470-0.9256) | 15.918 (15.5573-16.2787) | 4.973 (4.7345-5.2125) |
| Shandong | 1.3203 (1.2255-1.4151) | 11.682 (11.3864-11.8776) | 6.353 (6.1953-6.5107) |
| Liaoning | 0.9914 (0.9723-1.0104) | 6.382 (6.0943-6.6697) | 5.758 (5.3973-6.1187) |
| Henan | 1.0794 (1.0524-1.1064) | 11.616 (11.4145-11.8162) | 6.459 (6.3734-6.6166) |
| Ningxia | 0.8515 (0.7890-0.9139) | 14.509 (13.7663-15.2517) | 4.755 (4.5832-4.9268) |
| Shanxi | 0.5087 (0.4892-0.5283) | 11.201 (10.7591-11.6429) | 5.819 (5.6489-5.9891) |
| Hebei | 1.2397 (1.0895-1.3899) | 12.91 (12.649-13.171) | 6.544 (6.2962-6.6918) |
| Heilongjiang | 0.7864 (0.7605-0.8123) | 7.448 (7.1854-7.7106) | 5.538 (5.2718-5.8042) |
| Shannxi | 0.6855 (0.6541-0.717) | 10.115 (9.9321-10.2979) | 6.1516 (6.0809-6.2321) |
| Inner Mongolia | 1.0228 (1.0021-1.0435) | 9.525 (9.262-9.7878) | 5.7025 (5.5515-5.8535) |
| Jilin | 0.7667 (0.7292-0.7956) | 7.2026 (6.6134-7.7994) | 5.2854 (5.0902-5.4805) |

**Estimated values of transmission rates.** We fix the human indirect transmission rate

$\beta_{hw}$, and assume that $\beta_{hw} = 0.5$ for mainland China and 11 selected provinces with the highest incidence for human brucellosis. By using least-squares fitting method in DEDiscover software, the estimates of $\beta_s$, $\beta_{sw}$ and $\beta_h$ for these 11 provinces and mainland China can be obtained, and are shown in Table S3.

Table S3: Estimated values of $\beta_s$, $\beta_{sw}$ and $\beta_h$ with their 95% confidence intervals ($year^{-1}$).

| | $\beta_s$ 95% CI | $\beta_{sw}$ 95% CI | $\beta_h$ 95% CI |
|---|---|---|---|
| Mainland China | 0.5583 (0.5522-0.5643) | 0.1125 (0.1086-0.1186) | 0.0676 (0.0662-0.0691) |
| Xinjiang | 0.2395 (0.1602-0.3188) | 0.3037 (0.2832-0.3243) | 0.7291 (0.6525-0.8057) |
| Shandong | 0.5739 (0.3944-0.7534) | 0.3743 (0.3319-0.4176) | 0.9223 (0.7376-1.1070) |
| Liaoning | 0.1870 (0.1138-0.2602) | 0.2970 (0.2816-0.3123) | 0.0738 (0.0297-0.1180) |
| Henan | 0.2389 (0.1829-0.2950) | 0.3402 (0.3115-0.3689) | 0.4457 (0.3881-0.5032) |
| Ningxia | 0.3761 (0.2102-0.5420) | 0.2847 (0.2455-0.3240) | 0.4264 (0.2148-0.6380) |
| Shanxi | 0.3878 (0.3622-0.4134) | 0.0544 (0.0437-0.0650) | 0.9355 (0.9049-0.9661) |
| Hebei | 0.6213 (0.5381-0.7045) | 0.1847 (0.1550-0.2143) | 0.9295 (0.8963-0.9627) |
| Heilongjiang | 0.3781 (0.3176-0.4386) | 0.1311 (0.1167-0.1456) | 0.5256 (0.4561-0.5951) |
| Shaanxi | 0.4598 (0.4023-0.5173) | 0.0579 (0.0427-0.0731) | 0.0929 (0.0331-0.1565) |
| Inner Mongolia | 0.6275 (0.6030-0.6520) | 0.1437 (0.1344-0.1530) | 0.4638 (0.4401-0.4874) |
| Jilin | 0.5347 (0.3661-0.7034) | 0.1984 (0.1657-0.2311) | 0.4628 (0.3405-0.5851) |

## 2 Dynamical behavior

For the brucellosis model:

$$\begin{cases} \frac{dS_s}{dt} = bN_s - \beta_s S_s \frac{I_s}{N_s} - \beta_{sw} S_s f(W) - bS_s, \\ \frac{dI_s}{dt} = \beta_s S_s \frac{I_s}{N_s} + \beta_{sw} S_s f(W) - bI_s, \\ \frac{dW}{dt} = kI_s - \delta W, \\ \frac{dS_h}{dt} = b_h N_h + mI_h - \beta_h S_h \frac{I_s}{N_h} - \beta_{hw} S_h g(N_h, W) - d_h S_h, \\ \frac{dE_h}{dt} = \beta_h S_h \frac{I_s}{N_h} + \beta_{hw} S_h g(N_h, W) - \sigma E_h - d_h E_h, \\ \frac{dI_h}{dt} = \sigma E_h - pI_h - mI_h - d_h I_h, \\ \frac{dC_h}{dt} = pI_h - d_h C_h, \\ N_s = S_s + I_s, N_h = S_h + E_h + I_h + C_h. \end{cases} \tag{1}$$

As the last four equations are independent of the first three equations for system (1), we only need to consider the following system:

$$\begin{cases} \frac{dS_s}{dt} = bN_s - \beta_s S_s \frac{I_s}{N_s} - \beta_{sw} S_s f(W) - bS_s, \\ \frac{dI_s}{dt} = \beta_s S_s \frac{I_s}{N_s} + \beta_{sw} S_s f(W) - bI_s, \\ \frac{dW}{dt} = kI_s - \delta W. \end{cases} \tag{2}$$

For the general incidence rate $\beta_{sw}S_sf(W)$, nonnegative functions $f(W)$ is assumed to be differentiable with $W$, and thus solutions to system (2) with nonnegative initial conditions exist and are unique. Throughout we also assume the following properties of function $f(W)$, which are biologically reasonable:

(H1) Nonnegative function $f(W)$ vanishes when $W = 0$.

(H2) $f(W)$ is monotone nondecreasing with $W$.

(H3) $\frac{f(W)}{W}$ is monotone nonincreasing.

Notice that mass action incidence, saturating incidence, and standard incidence of system (2) satisfy assumptions (H1)-(H3). Omega limit sets of system (2) are contained in the following bounded region in the non-negative cone of $\mathbb{R}^3$:

$$X = \{(S_s, I_s, W)|S_s + I_s = N_s, 0 \le S_s, I_s \le N_s, 0 \le W \le \frac{kN_s}{\delta}\}.$$

Region $X$ is positively invariant with respect to system (2). It is obvious that any solution of system (2) with nonnegative initial values is nonnegative and system (2) has one disease-free equilibrium $P_0 = (N_s, 0, 0)$. We derive the basic reproduction number of system (2) by the next generation matrix formulated in Diekmann et al. [5, 6]. We order the infection variables first by disease state, only needing the vector $\mathbf{x} = (I_s, W)^T$. Considering the following auxiliary system:

$$\begin{cases} \frac{dI_s}{dt} = \beta_s S_s \frac{I_s}{N_s} + \beta_{sw} S_s f(W) - bI_s, \\ \frac{dW}{dt} = kI_s - \delta W, \end{cases} \tag{3}$$

Following the recipe from van den Driessche and Watmough [7] to obtain:

$$F = \begin{pmatrix} \beta_s & \beta_{sw} N_s f^{'}(0) \\ 0 & 0 \end{pmatrix}, V = \begin{pmatrix} b & 0 \\ -k & \delta \end{pmatrix}, V^{-1} = \begin{pmatrix} \frac{1}{b} & 0 \\ \frac{k}{b\delta} & \frac{1}{\delta} \end{pmatrix},$$

here $f^{'}(0)$ is the derivative of $f(W)$ with respect to $W$ at disease-free equilibrium. The basic reproduction number is defined as the spectral radius of the nonnegative matrix $FV^{-1}$; it is easy to obtain

$$\mathcal{R}_0 = \rho(FV^{-1}) = \frac{\beta_s}{b} + \frac{k\beta_{sw} N_s f^{'}(0)}{b\delta} = \mathcal{R}_0^i + \mathcal{R}_0^e,$$

where $\mathcal{R}_0^e = \frac{k\beta_{sw}N_sf^{'}(0)}{b\delta}$ and $\mathcal{R}_0^i = \frac{\beta_s}{b}$ are partial reproductive numbers due to environment-to-individual transmission and individual-to-individual transmission, respectively.

**Stability of the disease-free equilibrium.** System (2) is a cooperation system, and the following theorem shows that the disease-free equilibrium of system (2) is globally asymptotically stable in the region $X$.

**Theorem 2.1.** *Supposing that assumptions (H1)-(H3) hold, then the following conclusions hold for the system* (2). *(a) If* $\mathcal{R}_0 < 1$, *then the disease-free equilibrium* $P_0$ *of system* (2) *is globally asymptotically stable in the region* $X$. *(b) If* $\mathcal{R}_0 > 1$, *then the disease-free equilibrium* $P_0$ *of system* (2) *is unstable.*

*Proof.* One can obtain the following equation by using assumptions (H1) and (H3),

$$\frac{f(W)}{W} \leq \lim_{W\to 0}\frac{f(W)}{W} = \lim_{W\to 0}\frac{f(W)-f(0)}{W-0} = f^{'}(0),$$

which also means $f^{'}(0)W \geq f(W)$. Hence, for system (3), it is easy to obtain:

$$\frac{d\mathbf{x}}{dt} \leq (F-V)\mathbf{x}, \tag{4}$$

Let $\mathbf{b} \geq 0$ be the left eigenvector of the nonnegative matrix $V^{-1}F$ with respect to the eigenvalue $\rho(V^{-1}F) = \mathcal{R}_0$, that is, $\mathbf{b}^T V^{-1}F = \mathcal{R}_0\mathbf{b}^T$. Define the Lyapunov function:

$$L_1 = \mathbf{b}^T V^{-1}\mathbf{x}.$$

Then the derivative of $L$ along the system (3) is:

$$\frac{dL_1}{dt} = \mathbf{b}^T V^{-1}\mathbf{x}^{'} \leq \mathbf{b}^T V^{-1}(F-V)\mathbf{x} = \mathbf{b}^T V^{-1}F\mathbf{x} - \mathbf{b}^T\mathbf{x} \leq (\mathcal{R}_0 - 1)b^T\mathbf{x}.$$

If $\mathcal{R}_0 < 1$, then $\frac{dL_1}{dt} \leq 0$. Let:

$$\Psi = \{(S_s, I_s, W) \in X | \frac{dL_1}{dt} = 0\}.$$

If $\mathcal{R}_0 < 1$, $\frac{dL_1}{dt} = 0$ implies that $\mathbf{b}^T\mathbf{x} = 0$, thus $I_s = 0, W = 0$. Therefore, the largest invariant set of $\Psi$ is the singleton $P_0$. By LaSalle's invariance principle [8], $P_0$ is globally asymptotically stable in the region $X$ when $\mathcal{R}_0 < 1$.

If $\mathcal{R}_0 > 1$ and $\mathbf{x} > 0$, it follows that:

$$(\mathcal{R}_0 - 1)\mathbf{b}^T\mathbf{x} > 0, \tag{5}$$

There must exist $\frac{dL_1}{dt} > 0$ in a small enough neighborhood of $P_0$ in the interior of $X$. Therefore, solutions in the interior of $X$ sufficiently close to $P_0$ move away from $P_0$ provided $\mathcal{R}_0 > 1$, and thus $P_0$ is unstable. The proof is end. □

**The existence and stability of the endemic equilibrium.** Firstly, we will show the existence of the endemic equilibrium of system (2). It is assumed that $P^* = (S_s^*, I_s^*, W^*)$ is an endemic equilibrium of system (2), and satisfies the following equilibrium equations:

$$\begin{cases} bN_s = \beta_s S_s^* \frac{I_s^*}{N_s} + \beta_{sw} S_s^* f(W^*) + bS_s^*, \\ bI_s^* = \beta_s S_s^* \frac{I_s^*}{N_s} + \beta_{sw} S_s^* f(W^*), \\ kI_s^* = \delta W^*, \end{cases} \tag{6}$$

We have $W^* = \frac{kI_s^*}{\delta}$ and $S_s^* = N_s - I_s^*$. Therefore, the equilibrium of system (2) is equal to the following system:

$$bI_s^* = \beta_s(N_s - I_s^*)\frac{I_s^*}{N_s} + \beta_{sw}(N_s - I_s^*)f(\frac{kI_s^*}{\delta}). \tag{7}$$

Equation (7) has a zero solution $I_s^*=0$. Let,

$$F(I_s^*)=\beta_s(N_s-I_s^*)\frac{I_s^*}{N_s}+\beta_{sw}(N_s-I_s^*)f(\frac{kI_s^*}{\delta})-bI_s^*. \tag{8}$$

For equation (8), we can obtain:

$$\frac{dF(I_s^*)}{dI_s^*}=\beta_s-2\beta_s\frac{I_s^*}{N_s}+\beta_{sw}N_s\frac{k}{\delta}f^{'}(\frac{kI_s^*}{\delta})-\beta_{sw}f(\frac{kI_s^*}{\delta})-b.$$

Due to $F(0)=0$ and $F(N_s)=-bN_s<0$, so the sufficient and necessary condition for the existence of positive equilibrium of system (7) is:

$$\frac{dF(I_s^*)}{dI_s^*}|_{I_s^*=0}=\beta_s+\beta_{sw}N_s\frac{k}{\delta}f^{'}(0)-b=b(\mathcal{R}_0-1)>0.$$

Hence, we can conclude that for system (2) there at least exists an endemic equilibrium if $\mathcal{R}_0>1$.

**Theorem 2.2.** *Suppose that assumptions (H1)–(H3) hold. If $\mathcal{R}_0>1$, then the endemic equilibrium $P^*$ of system (2) is unique and globally asymptotically stable in the interior of $X$.*

*Proof.* Let $h(a)=1-a+\ln a$ for all $a>0$, then it is easy to verify that:

$$h(a)=1-a+\ln a\leq 0. \tag{9}$$

Let $D_1=S_s-S_s^*-S_s^*\ln\frac{S_s}{S_s^*}+I_s-I_s^*-I_s^*\ln\frac{I_s}{I_s^*}$ and $D_2=W-W^*-W^*\ln\frac{W}{W^*}$. Differentiating $D_1$ and $D_2$ with $t$ along solution curves of system (2) and using equilibrium equations (6) to simplify, one can obtain:

$$\begin{aligned}
\frac{dD_1}{dt} &= \left(1-\frac{S_s^*}{S_s}\right)S_s^{'}+\left(1-\frac{I_s^*}{I_s}\right)I_s^{'}\\
&= \left(1-\frac{S_s^*}{S_s}\right)\left(\beta_sS_s^*\frac{I_s^*}{N_s}-\beta_sS_s\frac{I_s}{N_s}+\beta_{sw}S_s^*f(W^*)-\beta_{sw}S_sf(W)+bS_s^*-bS_s\right)\\
&\quad+\left(1-\frac{I_s^*}{I_s}\right)\left(\beta_sS_s\frac{I_s}{N_s}-\beta_sS_s^*\frac{I_s^*}{N_s}\frac{I_s}{I_s^*}+\beta_{sw}S_sf(W)-\beta_{sw}S_s^*f(W^*)\frac{I_s}{I_s^*}\right)\\
&= \beta_{sw}S_s^*f(W^*)\left(\left(1-\frac{S_s^*}{S_s}\right)\left(1-\frac{S_sf(W)}{S_s^*f(W^*)}\right)+\left(1-\frac{I_s^*}{I_s}\right)\left(\frac{S_sf(W)}{S_s^*f(W^*)}-\frac{I_s}{I_s^*}\right)\right)\\
&\quad+bS_s^*\left(1-\frac{S_s}{S_s^*}\right)\left(1-\frac{S_s^*}{S_s}\right)+\beta_sS_s^*\frac{I_s^*}{N_s}\left(2-\frac{S_s^*}{S_s}-\frac{S_s}{S_s^*}\right)\\
&\leq \beta_{sw}S_s^*f(W^*)\left(2-\frac{I_s}{I_s^*}-\frac{S_s^*}{S_s}-\frac{S_sI_s^*f(W)}{S_s^*I_sf(W^*)}+\frac{f(W)}{f(W^*)}\right)\\
&\leq \beta_{sw}S_s^*f(W^*)\left(\frac{f(W)}{f(W^*)}-\ln\frac{f(W)}{f(W^*)}+\ln\frac{I_s}{I_s^*}-\frac{I_s}{I_s^*}\right)\\
&\leq \beta_{sw}S_s^*f(W^*)\left(\left(\frac{f(W)}{f(W^*)}-1\right)\left(1-\frac{f(W^*)W}{f(W)W^*}\right)+\frac{W}{W^*}-\ln\frac{W}{W^*}+\ln\frac{I_s}{I_s^*}-\frac{I_s}{I_s^*}\right)\\
&\leq \beta_{sw}S_s^*f(W^*)\left(\frac{W}{W^*}-\ln\frac{W}{W^*}+\ln\frac{I_s}{I_s^*}-\frac{I_s}{I_s^*}\right).
\end{aligned}$$

Through using inequality (9), we can obtain the second and third inequality with the following equations:

$$2-\frac{S_s^*}{S_s}-\frac{S_sI_s^*f(W)}{S_s^*I_sf(W^*)}\le -\ln\frac{S_s^*}{S_s}-\ln\frac{S_sI_s^*f(W)}{S_s^*I_sf(W^*)}=-\ln\frac{f(W)}{f(W^*)}+\ln\frac{I_s}{I_s^*}.$$

$$\begin{aligned}\frac{f(W)}{f(W^*)}-\ln\frac{f(W)}{f(W^*)} &= \frac{f(W)}{f(W^*)}+\ln\frac{f(W^*)W}{f(W)W^*}-\ln\frac{W}{W^*}\\ &\le \frac{f(W)}{f(W^*)}-1+\frac{f(W^*)W}{f(W)W^*}-\ln\frac{W}{W^*}\\ &= \left(\frac{f(W)}{f(W^*)}-1\right)\left(1-\frac{f(W^*)W}{f(W)W^*}\right)+\frac{W}{W^*}-\ln\frac{W}{W^*}.\end{aligned}$$

Following from assumptions (H2) and (H3), we have $\frac{f(W)}{f(W^*)}-1\ge 0, 1-\frac{f(W^*)W}{f(W)W^*}\le 0$ for $W^*\le W$, and $\frac{f(W)}{f(W^*)}-1<0, 1-\frac{f(W^*)W}{f(W)W^*}>0$ for $W^*>W$. Hence, one can obtain:

$$\left(\frac{f(W)}{f(W^*)}-1\right)\left(1-\frac{f(W^*)W}{f(W)W^*}\right)\le 0.$$

Similarly, we have the following equation:

$$\begin{aligned}\frac{dD_2}{dt} &= \left(1-\frac{W^*}{W}\right)W'=kI_s^*\left(1-\frac{W^*}{W}\right)\left(\frac{I_s}{I_s^*}-\frac{W}{W^*}\right)\\ &= kI_s^*\left(1-\frac{W}{W^*}+\frac{I_s}{I_s^*}-\frac{I_sW^*}{I_s^*W}\right)\le kI_s^*\left(-\frac{W}{W^*}+\ln\frac{W}{W^*}-\ln\frac{I_s}{I_s^*}+\frac{I_s}{I_s^*}\right).\end{aligned}$$

Hence, we can define the following Lyapunov function:

$$L_2=\frac{D_1}{\beta_{sw}S_s^*f(W^*)}+\frac{D_2}{kI_s^*}.$$

It follows that:

$$\begin{aligned}\frac{dL_2}{dt} &= \frac{1}{\beta_{sw}S_s^*f(W^*)}\frac{dD_1}{dt}+\frac{1}{kI_s^*}\frac{dD_2}{dt}\\ &\le \left(\frac{W}{W^*}-\ln\frac{W}{W^*}+\ln\frac{I_s}{I_s^*}-\frac{I_s}{I_s^*}\right)+\left(-\frac{W}{W^*}+\ln\frac{W}{W^*}-\ln\frac{I_s}{I_s^*}+\frac{I_s}{I_s^*}\right)=0.\end{aligned}$$

Moreover, the equality $\frac{dL_2}{dt}=0$ holds if and only if $S_s=S_s^*, I_s=I_s^*$ and $W=W^*$. Thus, $P^*$ is the only invariant set of system (2) in $\{(S_s,I_s,W)\in X:\frac{dL_2}{dt}=0\}$. By LaSalle's invariance principle [8], $P^*$ is globally asymptotically stable and unique in the interior of $X$ when $\mathcal{R}_0>1$. This completes the proof. □

**The endemic equilibrium of human population.** From previous analysis we know that the endemic equilibrium of system (2) is unique and globally asymptotically stable if $\mathcal{R}_0 > 1$. (1) The indirect transmission rate of human population is standard incidence, and $b_h > d_h$. When system (2) is stable, the last four equations of system (1) will become the following equations:

$$\begin{cases} \frac{dS_h}{dt} = b_h N_h + mI_h - \beta_h S_h \frac{I_s^*}{N_h} - \beta_{hw} S_h \frac{W^*}{N_h} - d_h S_h, \\ \frac{dE_h}{dt} = \beta_h S_h \frac{I_s^*}{N_h} + \beta_{hw} S_h \frac{W^*}{N_h} - \sigma E_h - d_h E_h, \\ \frac{dI_h}{dt} = \sigma E_h - pI_h - mI_h - d_h I_h, \\ \frac{dC_h}{dt} = pI_h - d_h C_h, \end{cases} \tag{10}$$

where $N_h = S_h + E_h + I_h + C_h$. For $\frac{dN_h}{dt} = (b_h - d_h)N_h$, it is easy to obtain $N_h(t) = N_h(0)e^{(b_h - d_h)t}$ and $\lim\limits_{t\to\infty} N_h(t) = \infty$. We make the following scaling transforms for system (10),

$$s_h = \frac{S_h}{N_h}, e_h = \frac{E_h}{N_h}, i_h = \frac{I_h}{N_h}, c_h = \frac{C_h}{N_h},$$

and we can obtain the following equations:

$$\begin{cases} \frac{ds_h}{dt} = b_h(1 - s_h) + mi_h - \beta_{hw} s_h \frac{W^*}{N_h} - \beta_h s_h \frac{I_s^*}{N_h}, \\ \frac{de_h}{dt} = \beta_{hw} s_h \frac{W^*}{N_h} + \beta_h s_h \frac{I_s^*}{N_h} - \sigma e_h - b_h e_h, \\ \frac{di_h}{dt} = \sigma e_h - pi_h - mi_h - b_h i_h, \\ \frac{dc_h}{dt} = pi_h - b_h c_h. \end{cases} \tag{11}$$

As $\lim\limits_{t\to\infty} N_h(t) = \infty$, we only need to consider the following limit system:

$$\begin{cases} \frac{ds_h}{dt} = b_h(1 - s_h) + mi_h, \\ \frac{de_h}{dt} = -\sigma e_h - b_h e_h, \\ \frac{di_h}{dt} = \sigma e_h - pi_h - mi_h - b_h i_h, \\ \frac{dc_h}{dt} = pi_h - b_h c_h. \end{cases} \tag{12}$$

For system (12), there is only a equilibrium $E_0 = (1, 0, 0, 0)$, and the equilibrium is globally asymptotically stable. Furthermore, let $P(t) = (S_h(t), E_h(t), I_h(t), C_h(t))$ be the positive equilibrium of system (10) at time $t$. We can obtain:

$$S_h(t) = \frac{N_h(t)}{\beta_h I_s^* + \beta_{hw} W^*}(\sigma + d_h)E_h(t), I_h(t) = \frac{\sigma}{p + m + d_h}E_h(t), C_h(t) = \frac{p}{d_h}I_h(t). \tag{13}$$

Taking the equations of system (13) into $N_h(t) = S_h(t) + E_h(t) + I_h(t) + C_h(t)$, we can obtain:

$$E_h(t) = \frac{N_h(t)}{\frac{(\sigma + d_h)(p + d_h) + md_h}{d_h(p + m + d_h)} + \frac{\sigma + d_h}{\beta_h I_s^* + \beta_{hw} W^*} N_h(t)}.$$

Hence,

$$\lim_{t\to\infty} E_h(t) = \lim_{t\to\infty} \frac{N_h(t)}{\frac{(\sigma + d_h)(p + d_h) + md_h}{d_h(p + m + d_h)} + \frac{\sigma + d_h}{\beta_h I_s^* + \beta_{hw} W^*} N_h(t)} = \frac{\beta_h I_s^* + \beta_{hw} W^*}{\sigma + d_h},$$

$$\lim_{t\to\infty} I_h(t) = \frac{\sigma}{p+m+d_h}\frac{\beta_h I_s^* + \beta_{hw}W^*}{\sigma + d_h}, \lim_{t\to\infty} C_h(t) = \frac{p\sigma}{d_h(p+m+d_h)}\frac{\beta_h I_s^* + \beta_{hw}W^*}{\sigma + d_h},$$

$$\lim_{t\to\infty} S_h(t) = \lim_{t\to\infty} \frac{N_h(t)}{\beta_h I_s^* + \beta_{hw}W^*}(\sigma + d_h)E_h(t) = \lim_{t\to\infty} N_h(t).$$

(2)

The indirect transmission rate of human population is saturating incidence or mass action incidence, and $b_h > d_h$. When system (2) is stable, the last four equations of system (1) will become the following equations:

$$\begin{cases} \frac{dS_h}{dt} = b_h N_h + mI_h - \beta_h S_h \frac{I_s^*}{N_h} - \beta_{hw} S_h g(W^*) - d_h S_h, \\ \frac{dE_h}{dt} = \beta_h S_h \frac{I_s^*}{N_h} + \beta_{hw} S_h g(W^*) - \sigma E_h - d_h E_h, \\ \frac{dI_h}{dt} = \sigma E_h - pI_h - mI_h - d_h I_h, \\ \frac{dC_h}{dt} = pI_h - d_h C_h, \end{cases} \tag{14}$$

Before discussing the details, we make the following scaling transforms for system (14):

$$s_h = \frac{S_h}{N_h}, e_h = \frac{E_h}{N_h}, i_h = \frac{I_h}{N_h}, c_h = \frac{C_h}{N_h}, \varphi = \beta_{hw} g(W^*).$$

We have,

$$\begin{cases} \frac{ds_h}{dt} = b_h(1 - s_h) + mi_h - \varphi s_h - \beta_{hw} s_h \frac{W^*}{N_h}, \\ \frac{de_h}{dt} = \varphi s_h + \beta_{hw} s_h \frac{W^*}{N_h} - \sigma e_h - b_h e_h, \\ \frac{di_h}{dt} = \sigma e_h - pi_h - mi_h - b_h i_h, \\ \frac{dc_h}{dt} = pi_h - b_h c_h. \end{cases} \tag{15}$$

Due to $\lim_{t\to\infty} N_h(t) = \infty$, so we only need to consider the following limit system:

$$\begin{cases} \frac{ds_h}{dt} = b_h(1 - s_h) + mi_h - \varphi s_h, \\ \frac{de_h}{dt} = \varphi s_h - \sigma e_h - b_h e_h, \\ \frac{di_h}{dt} = \sigma e_h - pi_h - mi_h - b_h i_h, \\ \frac{dc_h}{dt} = pi_h - b_h c_h. \end{cases} \tag{16}$$

Let $P_* = (s_h^*, e_h^*, i_h^*, c_h^*)$ be the positive equilibrium of system (16), we have:

$$s_h^* = \frac{b_h(p+m+b_h)(\sigma+b_h)}{(\varphi+d_h)(p+m+b_h)(\sigma+b_h) - m\sigma\varphi}, e_h^* = \frac{\varphi}{\sigma+b_h}s_h^*, i_h^* = \frac{\sigma}{p+m+b_h}e_h^*, c_h^* = \frac{p}{b_h}i_h^*.$$

For the stability of positive equilibrium $P_*$ of system (16), we have the following theorem.

**Theorem 2.3.** *The positive equilibrium $P_*$ of system* (16) *is globally asymptotically stable.*

*Proof.* To prove the local stability of positive equilibrium $P_*$ of system (16), all eigenvalues of the Jacobian matrix with system (16) at positive equilibrium $P_*$ should have negative real parts. The Jacobian matrix is:

$$J\mid_{P_*}=\begin{pmatrix} -(b_h+\varphi) & 0 & m & 0 \\ \varphi & -(\sigma+b_h) & 0 & 0 \\ 0 & \sigma & -(p+m+b_h) & 0 \\ 0 & 0 & p & -b_h \end{pmatrix}.$$

Hence, the corresponding characteristic equation of $J\mid_{E^*}$ is:

$$\begin{aligned} |z-J\mid_{P_*}\mid &= \begin{vmatrix} z+(b_h+\varphi) & 0 & -m & 0 \\ -\varphi & z+(\sigma+b_h) & 0 & 0 \\ 0 & -\sigma & z+(p+m+b_h) & 0 \\ 0 & 0 & -p & z+b_h \end{vmatrix} \\ &= (z+b_h)\begin{vmatrix} z+(b_h+\varphi) & 0 & -m \\ -\varphi & z+(\sigma+b_h) & 0 \\ 0 & -\sigma & z+(p+m+b_h) \end{vmatrix} \\ &= (z+b_h)(z^3+M_1z^2+M_2z+M_3)=0, \end{aligned}$$

where $M_1=\varphi+p+m+\sigma+3d_h>0, M_2=(\varphi+b_h)(p+m+b_h)+(\varphi+b_h)(\sigma+b_h)+(p+m+b_h)(\sigma+b_h)>0, M_3=(\varphi+b_h)(p+m+b_h)(\sigma+b_h)-m\sigma\varphi>0$, and $M_1M_2-M_3>0$.

Let $\Phi(z):=z^3+M_1z^2+M_2z+M_3=0$. By Routh–Herwitz criteria, all roots of $\Phi(z)$ have negative real parts, and hence the positive equilibrium $P_*$ of system (16) is locally asymptotically stable. Furthermore, the positive equilibrium $P_*$ of system (15) is globally asymptotically stable, because system (16) is linear. The proof is end. □

If the direct transmission rate of human population is not standard incidence, we can obtain the same conclusion with this situation.

*Remark* 2.4. For the transmission rate of human brucellosis, there are two kinds of situations. In the first situation both direct and indirect transmission rate of human population are standard incidence. In this situation, the human brucellosis cases will remain constant, but the proportion between infection cases and whole population will tend to 0 in the future when $\mathcal{R}_0>1$. In the second situation at least one of the direct and indirect transmission rate of human population is not standard incidence. In this situation, the human brucellosis cases will remain increasing, but the proportion between infected cases and whole population will tend to a constant in the future when $\mathcal{R}_0>1$.

# References


[1] Brucellosis: clinical manifestation. Available online: http://www.chinacdc.cn/jkzt/crb/blsjb/ (accessed on 2005.09.08).

[2] National Bureau of Statistics of China. *China Statistical Yearbook*; Human Population; China Statistics Press: Beijing, China, 2015.

[3] China Animal Husbandry Yearbook Editing Committee. *China Animal Husbandry Statistical Yearbook*; Statistical Information; China Agriculture Press: Beijing, China, 2015.

[4] Hou, Q.; Sun, X.; Zhang, J.; Liu, Y.; Wang, Y.; Jin, Z. Modeling the transmission dynamics of sheep brucellosis in Inner Mongolia Autonomous Region, China. *Math. Biosci.* **2013**, *242*, 51–58.

[5] Diekmann, O.; Heesterbeek, J.A.P.; Metz, J.A. On the definition and the computation of the basic reproduction ratio $R^0$ in models for infectious diseases in heterogeneous populations. *J. Math. Biol.* **1990**, *28*, 365–382.

[6] Diekmann, O.; Heesterbeek, J.; Roberts, M. The construction of next-generation matrices for compartmental epidemic models. *J R. Soc. Interface* **2009**, *7*, 873–885.

[7] Van den Driessche, P.; Watmough, J. Reproduction numbers and sub-threshold endemic equilibria for compartmental models of disease transmission. *Math. Biosci.* **2002**, *180*, 29–48.

[8] LaSalle, J.P. The stability of dynamical systems. in: regional conference series in applied mathematics, SIAM Philadelphia, 1976.
